# U-shaped relationship between early blood glucose and mortality in critically ill children

**DOI:** 10.1186/s12887-015-0403-y

**Published:** 2015-07-24

**Authors:** Yanhong Li, Zhenjiang Bai, Mengxia Li, Xueqin Wang, Jian Pan, Xiaozhong Li, Jian Wang, Xing Feng

**Affiliations:** Department of Nephrology, Suzhou, China; Institute of Pediatric Research, Suzhou, China; Pediatric Intensive Care Unit, Suzhou, China; Department of Neonatology, Children’s Hospital of Soochow University, 215003 Suzhou, China

**Keywords:** Critically ill children, Glucose, Hyperglycemia, Hypoglycemia, Intensive care, Mortality, Pediatric, Pediatric risk of mortality III

## Abstract

**Background:**

The aims of this study are to evaluate the relationship between early blood glucose concentrations and mortality and to define a ‘safe range’ of blood glucose concentrations during the first 24 h after pediatric intensive care unit (PICU) admission with the lowest risk of mortality. We further determine whether associations exist between PICU mortality and early hyperglycemia and hypoglycemia occurring within 24 h of PICU admission, even after adjusting for illness severity assessed by the pediatric risk of mortality III (PRISM III) score.

**Methods:**

This retrospective cohort study included patients admitted to PICU between July 2008 and June 2011 in a tertiary teaching hospital. Both the initial admission glucose values and the mean glucose values over the first 24 h after PICU admission were analyzed.

**Results:**

Of the 1349 children with at least one blood glucose value taken during the first 24 h after admission, 129 died during PICU stay. When analyzing both the initial admission and mean glucose values during the first 24 h after admission, the mortality rate was compared among children with glucose concentrations ≤65, 65-90, 90–110, 110–140, 140–200, and >200 mg/dL (≤3.6, 3.6–5.0, 5.0–6.1, 6.1–7.8, 7.8–11.1, and >11.1 mmol/L). Children with glucose concentrations ≤65 mg/dL (3.6 mmol/L) and >200 mg/dL (11.1 mmol/L) had significantly higher mortality rates, indicating a U-shaped relationship between glucose concentrations and mortality. Blood glucose concentrations of 110–140 mg/dL (6.1–7.8 mmol/L), followed by 90–110 mg/dL (5.0–6.1 mmol/L), were associated with the lowest risk of mortality, suggesting that a ‘safe range’ for blood glucose concentrations during the first 24 h after admission in critically ill children exists between 90 and 140 mg/dL (5.0 and 7.8 mmol/L). The odds ratios of early hyperglycemia (>140 mg/dL [7.8 mmol/L]) and hypoglycemia (≤65 mg/dL [3.6 mmol/L]) being associated with increased risk of mortality were 4.13 and 15.13, respectively, compared to those with mean glucose concentrations of 110–140 mg/dL (6.1–7.8 mmol/L) (*p* <0.001). The association remained significant after adjusting for PRISM III scores (*p* <0.001).

**Conclusions:**

There was a U-shaped relationship between early blood glucose concentrations and PICU mortality in critically ill children. Both early hyperglycemia and hypoglycemia were associated with mortality, even after adjusting for illness severity.

## Background

Both hyperglycemia and hypoglycemia are common complications of critical illnesses and are significantly associated with adverse outcomes. This has prompted clinical societies to recommend glucose control for critically ill patients [1–10]. However, tight glycemic control is not a standard practice in the pediatric intensive care unit (PICU) because of the lack of evidence for overall benefit and concerns about hypoglycemia [4, 11–14]. A recently published multicenter, randomized trial showed that tight glycemic control, with a target blood glucose range of 4.0 to 7.0 mmol/L (72 to 126 mg/dL), in critically ill children had no significant effect on major clinical outcomes. In that study, the incidence of hypoglycemia was higher with tight glucose control than with conventional glucose control [4]. One may raise a question as to whether it is possible that tight glycemic control significantly increased the risk of hypoglycemia and conferred no overall mortality benefit among critically ill children. In addition, a previous study conducted in critically ill adult patients demonstrated that there was a non-linear U-shaped relationship between mean glucose levels and ICU mortality in adult patients. Mean glucose levels <6.7 mmol/L (120 mg/dL) and >8.4 mmol/L (151 mg/dL) in the medical cohort and mean glucose levels <7.0 mmol/L (126 mg/dL) and >9.4 mmol/L (169 mg/dL) in the surgical cohort were associated with significantly increased ICU mortality. This results in a ‘safe range’ between approximately 7.0 and 9.0 mmol/L (126 and 162 mg/dL) in the mixed cohort of surgical and medical patients [15]. However, whether these findings apply to critically ill children remains unclear, and little is known about the level of the ‘safe range’ in children.

Furthermore, despite the publication of previous studies that investigated the association of hyperglycemia and hypoglycemia with mortality in the pediatric population [2, 16–23], information on the importance of early hyperglycemia and hypoglycemia in children is limited [8, 24, 25]. The association of early hyperglycemia, as opposed to serial measurements of glucose during the PICU stay, with mortality in critically ill children is controversial [8, 24, 25].

The aims of this study are to evaluate the relationship between early blood glucose concentrations and PICU mortality and to define a ‘safe range’ of blood glucose concentrations during the first 24 h after PICU admission with the lowest risk of mortality. We further determine whether associations of PICU mortality with early hyperglycemia and hypoglycemia occurring within 24 h of PICU admission exist even after adjusting for the severity of illness as assessed by the score of the pediatric risk of mortality (PRISM) III in critically ill children.

## Methods

### Study design

The study was designed as a retrospective cohort analysis of all children admitted to the PICU during the period of July 2008 to June 2011. The setting for this study was a 15-bed PICU with both medical and surgical patients in a university-affiliated tertiary children’s hospital. The criteria for PICU admission were adopted from guidelines published by the American Academy of Pediatrics [26]. We excluded patients with a clinical diagnosis of diabetes mellitus, patients who did not have a blood glucose measurement taken during the first 24 h after admission, and patients who were unexpectedly discharged or transferred to another hospital. For children with multiple PICU admissions within a single hospital stay, only the last admission was included in the study. The Institutional Review Board of the Children’s Hospital of Soochow University approved the study. Informed consent was not required, because all data were collected retrospectively.

### Clinical and laboratory data

Clinical and laboratory data from the day of admission were collected and included age, gender, admission diagnosis, routine hematological tests, a serum biochemical profile, and an arterial blood gas analysis. Clinical status, medications given, and therapeutic interventions administered were recorded daily until PICU discharge or death.

### The PRISM III score

The PRISM III score was calculated according to the methods described in the original studies [27, 28], and in accordance with our previous study [29].

### Clinical outcome

The primary outcome measure was PICU mortality rate, which was defined as deaths occurring during the PICU stay. Survivors in the study were discharged to home or transferred to another department in our hospital.

### Glucose parameters and measurements

During the study period, there was no standardized practice in place for monitoring blood glucose; therefore, all glucose values were obtained under the discretion of the treating physicians. All of the children in the study had at least one blood glucose value measured in the first 24 h after admission.

Whole blood glucose concentrations were measured on arterial or venous samples using an automatic biomedical blood gas analyzer (CCX, NOVA Biomedical, Waltham, MA, USA) with a colorimetric assay. System correlation calibration was performed every 6 months. The coefficient of variation was 2.8 % at the low level and 2.5 % at the high level of blood glucose concentration. The concentration of blood glucose was expressed in milligram per deciliter (mg/dL) in the study.

### Treatment protocol

Protocolized insulin infusion therapy was not utilized. Insulin administration and nutritional support route were determined by the treating physicians.

### Data management, interpretation, and analysis

To evaluate the relationship between early blood glucose concentrations and PICU mortality and to define a ‘safe range’ for blood glucose concentrations during the first 24 h after PICU admission, both the initial admission glucose value and the mean glucose value over the first 24 h after PICU admission were analyzed. The initial admission glucose values and the mean glucose values over the first 24 h of admission were divided into 6 strata based on cutoff values of 65, 90, 110, 140, and 200 mg/dL (3.6, 5.0, 6.1, 7.8, and 11.1 mmol/L), which were established according to clinical observational and interventional pediatric studies [16, 19, 22]. PICU mortality was calculated for each stratum, and the stratum with the lowest mortality incidence was used as a reference.

There are no specific criteria for defining hyperglycemia or hypoglycemia in critically ill children. Based on previous studies conducted in children, hyperglycemia was defined as a mean blood glucose concentration >140 mg/dL (7.8 mmol/L) [7, 19], and hypoglycemia was defined as a mean glucose concentration ≤65 mg/dL (3.6 mmol/L) [16, 19, 22]. Early hyperglycemia and hypoglycemia were defined as hyperglycemia and hypoglycemia occurring within the first 24 h after admission to the PICU. Glycemic variability was defined as any patient who had both a hyperglycemic measurement and a hypoglycemic measurement during the first 24 h after PICU admission [2].

### Statistical analysis

Statistical analyses were performed by using SPSS 13.0. The data are presented as mean ± SD or median and interquartile range (IQR), depending on the distribution of the data. For non-normally distributed variable, the Mann-Whitney U test was used to compare two groups, and the Kruskal-Wallis H test was used to determine the differences among groups.

The glucose values were divided into six strata, and PICU mortality was calculated for each stratum. The stratum with the lowest mortality incidence was used as a reference. The chi-square test or Fisher’s exact test was used to compare glucose strata. Subsequently, univariate binary logistic regression analyses were conducted to calculate the odds ratio (OR) for PICU mortality. Glucose strata were coded as categorical variables with the stratum with the lowest risk of mortality used as a reference. Multivariate binary logistic regression analyses were performed to investigate whether blood glucose concentrations were independently associated with PICU mortality after adjusting for age, gender, and severity of illness as assessed by the PRISM III score. To determine whether there was significant collinearity between glucose concentrations and PRISM III scores, collinearity diagnostics were performed using variance inflation factor (VIF) and tolerance values. All probability values are two-sided. Differences with *p* values <0.05 were considered to be statistically significant.

## Results

### Patient characteristics

In total, 1486 children admitted to the PICU during the study period were eligible for this study. One hundred and thirty children were excluded: 51 patients who did not have glucose measurements taken during the first 24 h after PICU admission, 62 who were unexpectedly discharged due to economic reasons, and 17 who were transferred to another hospital. Seven children had multiple PICU admissions within a single hospital stay, only their last admission was included in the analysis. Therefore, this study included 1349 critically ill children, including 1277 children with a medical admission diagnosis and 72 with a surgical admission diagnosis. Admission diagnoses included respiratory diseases (42.4 %), neurological diseases (22.8 %), gastrointestinal diseases (7.0 %), trauma or postoperative acute care (5.4 %), cardiovascular diseases (4.7 %), sepsis (4.0 %), hematologic/oncologic diseases (3.6 %), poisoning (2.6 %), and others (7.5 %). Of the 1349 children analyzed in the study, 129 (9.6 %) died during their PICU stay. The median time from PICU admission to death was 48 h (IQR, 24 to 96) after admission.

A total of 2010 blood glucose measurements were analyzed, including 1665 values from arterial blood samples and 345 from venous blood samples. The median blood glucose concentration at admission to the PICU in critically ill children was 127 mg/dL (7.1 mmol/L) (IQR, 100 to 180 mg/dL [5.6 to 10.0 mmol/L]). Admission glucose concentrations were >140 mg/dL (7.8 mmol/L) in 40.2 %, >180 mg/dL (10.0 mmol/L) in 25.0 %, and >200 mg/dL (11.1 mmol/L) in 19.3 % of children. However, only 2.4 % received insulin therapy (0.1u/kg.h) in the first 24 h after PICU admission to maintain blood glucose concentration <150 mg/dL (8.3 mmol/L). Admission glucose concentrations were ≤90 mg/dL (5.0 mmol/L) in 15.0 % and ≤65 mg/dL (3.6 mmol/L) in 3.6 % of children. Fifteen children (1.1 %) developed glycemic variability during the first 24 h after admission. Comparisons of demographic and clinical characteristics between critically ill children who did and did not survive are displayed in Table 1.

**Table 1:** Comparison of demographic and clinical characteristics between critically ill children who did and did not survive

Characteristics	Survivors (*n* =1220)	Non-survivors (*n* =129)	*P* value
Age, months	12.0 [3.0–36.0]	11.0 [3.0–36.0]	0.428
Gender, male, n	784 (64.3)	70 (54.3)	0.027
PRISM III score	3 [0–6]	12 [5–24]	<0.001
Admission glucose in mg/dL	126 [100–171]	176 [97–374]	<0.001
Admission glucose in mmol/L	7.0 [5.6–9.5]	9.8 [5.4–20.8]	<0.001
Use of insulin^a^, n	13 (1.1)	20 (15.5)	<0.001
Use of steroid^a^, n	583 (47.8)	55 (42.6)	0.308
Mechanical ventilation^b^, n	205 (16.8)	78 (60.5)	<0.001
MODS ≥3^c^, n	64 (5.2)	61 (47.3)	<0.001

Values are median [interquartile range]. Numbers in parentheses denote percentages

*MODS* multi-organ dysfunction syndrome, *PRISM III* pediatric risk of mortality III

^a^Administration during the first 24 h after PICU admission. ^b^Administration during PICU stay. ^c^MODS developed during PICU stay

### Relationship between blood glucose at admission and PICU mortality

**Figure 1:**
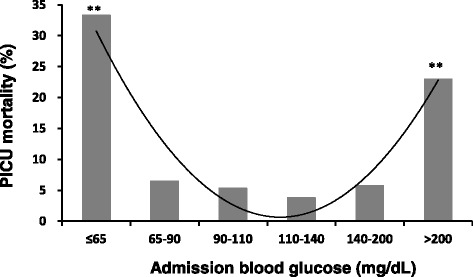
PICU Mortality rates according to different admission glucose cutoff values. *PICU*, pediatric intensive care unit. Curve represents a polynomial trendline. *p* value: comparison to children with admission blood glucose of 110 to 140 mg/dL (6.1 to 7.8 mmol/L). **p <0.01. Probability values: Chi-square test

The children were divided into 6 strata based on their admission glucose values. Comparisons of demographic and clinical characteristics among the strata are displayed in Table 2. Admission blood glucose ranges for each stratum and the corresponding PICU mortality rates are displayed in Fig. 1. The results reveal a U-shaped curve relationship between glucose concentrations at admission and PICU mortality in critically ill children. The lowest PICU mortality was observed in the stratum with glucose concentrations of 110–140 mg/dL (6.1–7.8 mmol/L) (3.8 %), followed by the stratum with glucose concentrations of 90–110 mg/dL (5.0–6.1 mmol/L) (5.3 %). There was no significant difference in the mortality rate between the two strata (3.8 % vs. 5.3 %, *p* =0.428). Children in the lowest stratum, with glucose concentrations ≤65 mg/dL (3.6 mmol/L), and in the highest stratum, with glucose concentrations >200 mg/dL (11.1 mmol/L), had significantly higher mortality rates compared to those with glucose concentrations of 110–140 mg/dL (6.1–7.8 mmol/L) (*p* <0.001).

**Table 2:** Comparison of demographic and clinical characteristics among children with different values of admission blood glucose

Blood glucose, mg/dL (mmol/L)	≤65 (≤3.6)	65–90 (3.6–5.0)	90–110 (5.0–6.1)	110–140 (6.1–7.8)	140–200 (7.8–11.1)	>200 (>11.1)	*P* value
N	48 (3.6)	155 (11.5)	262 (19.4)	342 (25.4)	281 (20.8)	261 (19.3)	
Age, months	8.0 [2.0–30.0]	8.0 [2.0–24.0]	11.5 [3.0–37.5]	9.0 [3.0–32.3]	12.0 [5.0–48.0]	12.0 [4.0–36.0]	0.002
Gender, male, n	27 (56.3)	103 (66.5)	179 (68.3)	225 (65.8)	179 (63.7)	141 (54.0)	0.013
PRISM III score	5 [2–12]	2 [0–5]	2 [0–5]	3 [0–6]	3 [0–6]	7 [4–14]	<0.001
Mechanical ventilation^a^, n	14 (29.8)	20 (12.8)	30 (11.5)	54 (15.8)	60 (21.4)	95 (36.4)	<0.001
MODS ≥3^b^, n	13 (27.7)	9 (5.8)	7 (2.7)	20 (5.8)	16 (5.7)	60 (23.0)	<0.001
PICU mortality, n	16 (34.0)	10 (6.4)	14 (5.3)	13 (3.8)	16 (5.7)	60 (23.0)	0.003

Values are median [interquartile range]. Numbers in parentheses denote percentages

*MODS* multi-organ dysfunction syndrome, *PRISM III* pediatric risk of mortality III

^a^Administration during PICU stay. ^b^MODS developed during PICU stay

### Relationship between mean glucose concentrations and PICU mortality

Of all of the children studied, 47.0 % had two and 2.0 % had three blood glucose measurements taken in the first 24 h after they were admitted to the PICU. There was no difference in the median number of glucose measurements during the first 24 h after admission between surviving and non-surviving children (*p* =0.137).

**Figure 2:**
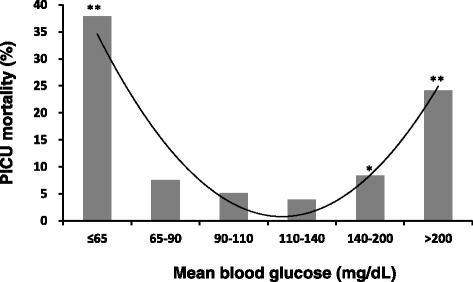
PICU Mortality rates according to different mean glucose cutoff values. *PICU,* pediatric intensive care unit. Curve represents a polynomial trendline. *p* value: comparison to children with mean blood glucose of 110 to 140 mg/dL (6.1 to 7.8 mmol/L). *p <0.05, **p <0.01. Probability values: Chi-square test

**Figure 3:**
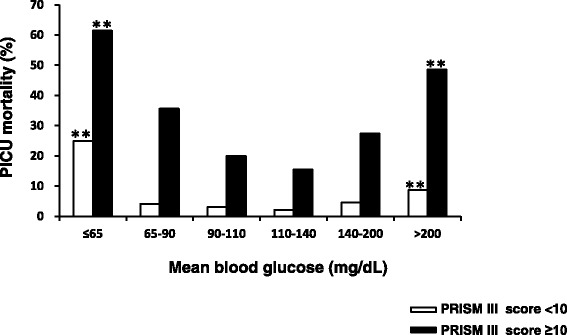
PICU Mortality rates according to different mean glucose cutoff values, stratified by PRISM III score. *PICU,* pediatric intensive care unit; *PRISM III,* pediatric risk of mortality III. *p* value: comparison to children with mean blood glucose of 110 to 140 mg/dL (6.1 to 7.8 mmol/L). **p <0.01. Probability values: Chi-square test or Fisher’s exact test

The mean glucose concentrations during the first 24 h after PICU admission were analyzed to define a ‘safe range’ for blood glucose in the first 24 h after admission. Figure 2 demonstrates the U-shaped curve relationship between the mean glucose concentrations in the first 24 h after admission and PICU mortality in critically ill children, showing high mortality in children with mean glucose concentrations >200 mg/dL (11.1 mmol/L) and in those with mean glucose concentrations ≤65 mg/dL (3.6 mmol/L). Additional analyses stratified children by the severity of their illnesses as assessed by PRISM III scores in Fig. 3. In both subgroups of children with PRISM III scores <10 and ≥10, children with mean blood glucose concentrations >200 mg/dL (11.1 mmol/L) and children with mean blood glucose concentrations ≤65 mg/dL (3.6 mmol/L) had significantly increased PICU mortality rates compared to those with mean blood glucose concentrations of 110 to 140 mg/dL (6.1–7.8 mmol/L).

In addition, children with mean glucose concentrations of 90–110 mg/dL (5.0–6.1 mmol/L) (5.1 %) and 110–140 mg/dL (6.1–7.8 mmol/L) (3.9 %) during the first 24 h after admission had low PICU mortality rates. Children with mean blood glucose concentrations ≤90 mg/dL (5.0 mmol/L) (14.0 % vs. 3.9 %, *p* <0.001) and >140 mg/dL (7.8 mmol/L) (14.2 % vs. 3.9 %, *p* <0.001) had significantly increased PICU mortality rates compared to those with mean blood glucose concentrations of 110 to 140 mg/dL (6.1 to 7.8 mmol/L). These differences remained significant after adjusting for PRISM III scores (*p* <0.05). This results in a ‘safe range’ for blood glucose concentrations of approximately 90 to 140 mg/dL (5.0–7.8 mmol/L) during the first 24 h after PICU admission in critically ill children.

Because PRISM III scores include the glucose level measured in the first 24 h after PICU admission, collinearity diagnostics were performed using VIF and tolerance values before multivariate logistic regression analysis was performed. VIF and tolerance values of 1.128 and 0.87, respectively, indicate the absence of significant collinearity between glucose concentrations and PRISM III scores.

### Association of PICU mortality with early hyperglycemia and hypoglycemia

Hyperglycemia was prevalent among children in the PICU; 541 children (40.1 %) were in a hyperglycemic state, defined as a mean glucose concentration >140 mg/dL (7.8 mmol/L) in the first 24 h after admission. Hypoglycemia, defined as a mean blood glucose concentration ≤65 mg/dL (3.6 mmol/L), occurred in 37 (2.7 %) of the children. A mean blood glucose concentration of 110 to 140 mg/dL (6.1 to 7.8 mmol/L) was used as a reference. Both early hyperglycemia and hypoglycemia were coded as a categorical variable and were significantly associated with PICU mortality in a univariate binary logistic regression analysis.

The OR for early hyperglycemia being associated with an increased risk of mortality was 4.13 (95 % CI, 2.30–7.41; *p* <0.001). The association remained significant after adjusting for age, gender, and PRISM III scores using a multivariate binary logistic regression analysis (OR =2.06; 95 % CI, 1.07–3.96; *p* =0.031). We further evaluated the association between severe and mild hyperglycemia and PICU mortality. A mean blood glucose concentration >200 mg/dL (11.1 mmol/L) was considered as severe hyperglycemia, and a level of 140–200 mg/dL (7.8–11.1 mmol/L) as mild hyperglycemia. As reported in Table 3, both severe and mild hyperglycemia were associated with increased PICU mortality compared to mean blood glucose concentrations of 110 to 140 mg/dL (6.1–7.8 mmol/L). However, only severe hyperglycemia remained associated with PICU mortality after adjusting for age, gender, and PRISM III scores (OR =2.25; 95 % CI, 1.17–4.36; *p* =0.016).

**Table 3:** Association of PICU mortality with early hyperglycemia and hypoglycemia: comparison to children with mean blood glucose of 110 to 140 mg/dL*

Mean Glucose, mg/dL (mmol/L)	OR^a^	95 % CI	*P* value
>200 (11.1)^a^	7.86	4.21–14.66	<0.001^d^
140–200 (7.8–11.1)^b^	2.25	1.17–4.36	0.016^e^
110–140 (6.1–7.8)	1		N.A.
90–110 (5.0–6.1)	1.37	0.64–2.92	0.416
65–90 (3.6–5.0)	1.90	0.82–4.38	0.133
≤65 (3.6)^c^	15.13	6.45–35.49	<0.001^d^

*The mean glucose concentration of the first 24 h of admission >140 mg/dL was defined as early hyperglycemia; mean glucose concentration ≤65 mg/dL was defined as early hypoglycemia

Glucose strata were coded as a categorical variable with the mean glucose concentration of 110–140 mg/dL as a reference

*CI* confidence interval, *OR* odds ratio, *PICU* pediatric intensive care unit

^a^severe hyperglycemia. ^b^mild hyperglycemia. ^c^hypoglycemia. ^d^The association remained significant after adjusting for age, gender, and PRISM III scores. ^e^The association did not remain significant after adjusting for age, gender, and PRISM III scores

The OR for PICU mortality being associated with hypoglycemia was 15.13 (95 % CI, 6.45–35.49; *p* <0.001) compared to those with mean blood glucose concentrations of 110 to 140 mg/dL (6.1–7.8 mmol/L). The association remained significant after adjusting for age, gender, and PRISM III scores using a multivariate binary logistic regression analysis (OR =12.68; 95 % CI, 4.48–35.88; *p* <0.001).

### Association of PICU mortality with early hyperglycemia and hypoglycemia, stratified by illness severity

The association of PICU mortality with both early hyperglycemia (>140 mg/dL [7.8 mmol/L]) and hypoglycemia (≤65 mg/dL [3.6 mmol/L]) remained significant after adjusting for illness severities as assessed by PRISM III scores (hyperglycemia: OR =2.13; 95 % CI, 1.11–4.10; *p* =0.023; hypoglycemia: OR =12.01; 95 % CI, 4.42–32.62; *p* <0.001).

In addition, as displayed in Table 4, severe hyperglycemia (>200 mg/dL [11.1 mmol/L]) and hypoglycemia (≤65 mg/dL [3.6 mmol/L]) were consistently associated with an increased risk of PICU mortality in both subgroups of children with PRISM III scores <10 and ≥10.

**Table 4:** Association of PICU mortality with early hyperglycemia and hypoglycemia, stratified by PRISM III score: comparison to children with mean blood glucose of 110 to 140 mg/dL*

	Mean glucose, mg/dL (mmol/L)	OR	95 % CI	*P* value
PRISM III <10	>200 (11.1)^a^	4.17	1.58–11.02	0.005
	140–200 (7.8–11.1)^b^	2.13	0.84–5.43	0.115
	110–140 (6.1–7.8)	1		N.A.
	90–110 (5.0–6.1)	1.48	0.53–4.14	0.599
	60–90 (3.6–5.0)	1.80	0.56–5.79	0.338
	≤65 (3.6)^c^	14.67	4.46–48.19	<0.001
PRISM III ≥10	>200 (11.1)^a^	5.14	2.04–12.98	<0.001
	140–200 (7.8–11.1)^b^	2.05	0.75–5.66	0.217
	110–140 (6.1–7.8)	1		N.A.
	90–110 (5.0–6.1)	1.36	0.35–5.29	0.725
	60–90 (3.6–5.0)	3.02	0.78–11.73	0.133
	≤65 (3.6)^c^	8.69	2.19–34.45	0.002

*The mean glucose concentration of the first 24 h of admission >140 mg/dL was defined as early hyperglycemia; mean glucose concentration ≤65 mg/dL was defined as early hypoglycemia

Glucose strata were coded as a categorical variable with the mean glucose concentration of 110–140 mg/dL as a reference

*CI* confidence interval, *OR* odds ratio, *PICU* pediatric intensive care unit, *PRISM III* pediatric risk of mortality III

^a^severe hyperglycemia. ^b^mild hyperglycemia. ^c^hypoglycemia

## Discussion

This study provides data on early blood glucose concentrations in critically ill children and demonstrates that there is a U-shaped relationship between blood glucose concentrations measured during the first 24 h after PICU admission and PICU mortality. Blood glucose concentrations of 110–140 mg/dL (6.1–7.8 mmol/L), followed by 90–110 mg/dL (5.0–6.1 mmol/L), were associated with the lowest OR for mortality, suggesting that a ‘safe range’ for blood glucose concentrations exists between 90 and 140 mg/dL in the first 24 h after PICU admission in critically ill children.

The U-shaped relationship between blood glucose concentrations at admission and PICU mortality, with increased mortality in the lower and upper levels in this study, is in line with recently published findings conducted on critically ill adult patients [15]. The optimal glucose concentrations in our study are somewhat lower than the results from the cohort of adult patients. This is possibly due to differences between children and adults. The normal range of blood glucose level associated with reduced morbidity and mortality decreases with age in non-neonatal children [30]. Another difference is the low percentage of our patients admitted with a surgical diagnosis.

The other major findings in this study were that both early hyperglycemia and hypoglycemia occurring within 24 h of PICU admission were associated with increased PICU mortality rates, even after adjusting for age, gender and illness severity in this population of critically ill children. Numerous studies have demonstrated that hyperglycemia is associated with increased mortality rates in children [2, 16–18, 23]. To our knowledge, a limited number of studies verified the use of early hyperglycemia as a prognostic predictor in critically ill children [8, 24, 25]. Our results are in agreement with a study by Faustino, who reported that the relative mortality risk increased for PICU patients with glucose concentrations >150 mg/dL (8.3 mmol/L) in the first 24 h after admission [8].

The discrepancy between our data and the data from another study performed in children admitted to the PICU can most likely be attributed to the difference in the cutoff value for defining hyperglycemia [25]. Our study showed that hyperglycemia, defined as a blood glucose concentration >140 mg/dL (7.8 mmol/L), was significantly associated with a higher PICU mortality rate. However, In the previous study, they used a blood glucose concentration of 200 mg/dL (11.1 mmol/L) as the threshold for differentiating high glucose concentrations from normal glucose concentrations, and concluded that hyperglycemia within 24 h of PICU admission was not independently associated with increased mortality. The normal glucose (<200 mg/dL [11.1 mmol/L]) group included patients with glucose concentrations of 60–200 mg/dL (3.3–11.1 mmol/L) [25]. It is possible that blood glucose concentrations between 60 and 90 mg/dL (3.3 and 5.0 mmol/L) or between 140 and 200 mg/dL (7.8 and 11.1 mmol/L) in children could significantly increase their risk of mortality and confer no advantage.

In the present study, 2.7 % of the patients had mean glucose concentrations ≤65 mg/dL (3.6 mmol/L) in the first 24 h after PICU admission. It is well known that the developing brains of children, especially infants, are more vulnerable to hypoglycemia [13, 22]. The implementation of tight glucose control is often tempered by concerns of inducing hypoglycemia [11, 13, 31, 32]. The definition of hypoglycemia is not consistent and varies in clinical practice for non-diabetic children [22]. Most have defined hypoglycemia as blood glucose concentrations <40 or <60–65 mg/dL (<2.2 or <3.3–3.6 mmol/L), while some have reported a definition of glucose concentrations <80 mg/dL (4.4 mmol/L) [16, 20–22, 33]. Although a few studies have concluded, based on point-of-care glucose measurements during PICU stays, that hypoglycemia is associated with increased mortality in critically ill children [16, 20–22], we were first to demonstrate that hypoglycemia occurring within 24 h of PICU admission was significantly associated with higher PICU mortality rates. Moreover, the association was independent of age, gender, and illness severity as assessed by PRISM III scores.

Our study has several limitations. The first is the weakness that is inherent in retrospective studies. There was no standard technique or protocol for collecting or analyzing blood glucose samples. Second, both arterial and venous whole blood glucose concentrations were analyzed in this study. It has been found that there were no differences between median arterial (161 mg/dL [8.9 mmol/L]) and venous (162 mg/dL [9.0 mmol/L]) whole blood glucose concentrations in critical ill patients after cardiac surgery, which suggests that venous blood may be a good alternative to arterial blood for glucose measurements [34]. However, differences between glucose values drawn via different sample sites should be considered. Third, our findings may not be generalizable to critically ill children receiving aggressive glucose control. In our study, 25 % of the patients had an initial blood glucose concentration >180 mg/dL (10.0 mmol/L); however, only 2.4 % received insulin therapy in the first 24 h after they were admitted to the PICU. Further multicenter studies will be needed to confirm our findings and improve the generalizability of these results. Finally, we were unable to include data regarding medications or nutrition in the analysis to assess their effect on the relationship between blood glucose concentrations and mortality, as we did not have information concerning these data for all of the patients during their transfers to the PICU.

## Conclusions

Our study indicates that there is a U-shaped relationship between early blood glucose concentrations and PICU mortality. Blood glucose concentrations of 110–140 mg/dL (6.1–7.8 mmol/L), followed by 90–110 mg/dL (5.0–6.1 mmol/L), were associated with the lowest OR for mortality, suggesting a ‘safe range’ of blood glucose concentrations of between 90 and 140 mg/dL (5.0–7.8 mmol/L) in the first 24 h after PICU admission in critically ill children. Both early hyperglycemia and hypoglycemia occurring within 24 h of PICU admission were significantly associated with mortality in critically ill children, even after adjusting for age, gender, and illness severity. Further studies are necessary to define the ‘safe range’ in randomized prospective clinical trials.

## Abbreviations

CI: confidence interval
IQR: interquartile range
MODS: multi-organ dysfunction syndrome
OR: odds ratio
PICU: pediatric intensive care unit
PRISM III: Pediatric Risk of Mortality III
VIF: variance inflation factor
